# Interleukin-6 in Daily Use in the Intensive Care Unit: Does It Change the Patients’ Outcome and Antimicrobial Prescription? An Explorative Study

**DOI:** 10.3390/life16040590

**Published:** 2026-04-01

**Authors:** Tobias Bexten, Rumen Kasabov, Stefan Bushuven, Anne Kamphausen, Verena Schneider-Lindner, Holger A. Lindner

**Affiliations:** 1 Department for Interdisciplinary Intensive Medicine and Intermediate Care, Helios Dr. Horst Schmidt Hospital Wiesbaden, 65199 Wiesbaden, Germany; rumen.kasabov@helios-gesundheit.de; 2 Department of Anesthesiology and Critical Care, University of Freiburg Medical Center, 79106 Freiburg, Germany; stefan.bushuven@notis-ev.de; 3 Department for Anesthesiology and Surgical Intensive Care Medicine, Paracelsus Medical University, Nuremberg General Hospital, 90419 Nuremberg, Germany; anne.kamphausen@klinikum-nuernberg.de; 4 Department of Anesthesiology, Intensive Care Medicine and Pain Medicine, Medical Faculty Mannheim of Heidelberg University, 68167 Mannheim, Germany; verena.schneider-lindner@medma.uni-heidelberg.de (V.S.-L.); holger.lindner@medma.uni-heidelberg.de (H.A.L.)

**Keywords:** IL-6, CRP, procalcitonin, biomarkers, defined daily dose (DDD), antimicrobial stewardship, RDD, ICU outcomes

## Abstract

Background: Interleukin-6 (IL-6) rises rapidly during systemic inflammation and is used in some ICUs as a daily infection marker. Whether routine IL-6 monitoring affects patient outcomes or antimicrobial use, compared with standard biomarkers CRP/PCT, remains unclear. Methods: This retrospective two-step study was conducted at a tertiary interdisciplinary ICU in Wiesbaden, Germany. Step 1 (pilot cohort) compared two 2-month periods with routine IL-6 versus CRP/PCT testing to identify differences and generate assumptions. Step 2 (extended cohort) compared two consecutive 12-month periods before and after discontinuation of IL-6 testing. After matching for disease severity and specialty, endpoints included ICU length of stay, ventilation hours, mortality, and antimicrobial use measured as defined daily doses (DDD), and recommended daily doses (RDD) per 100 patient-days. Results: Results: In the pilot cohort (n = 221), there were no significant differences between the IL-6 and CRP/PCT groups in terms of anti-infective therapy or ventilation hours. In the extended cohort (n = 5146), case-matched analyses showed no significant group differences in ICU length of stay, ventilation hours, or mortality between groups. Antimicrobial consumption was higher when IL-6 was used: DDD (16.8% increase, rate ratio RR = 1.17, 95% CI (1.14, 1.19), *p* < 0.001) and RDD (11.6% increase, RR = 1.12, 95% CI (1.09, 1.14), *p* < 0.001). Conclusions: In this exploratory study, routine IL-6 testing was not associated with improved outcomes but might be linked to increased antimicrobial consumption.

## 1. Introduction

Critically ill patients in intensive care units (ICUs) are at high risk of developing infections or are admitted due to infections, such as ventilator-associated pneumonia (VAP) or surgical-site infections, that contribute to increased morbidity and mortality [1,2]. VAP is among the leading infections in the ICU and is sometimes challenging to diagnose [3] due to concomitant organ dysfunction, overlapping symptoms, and underlying diseases. Inflammatory biomarkers such as C-reactive protein (CRP) and procalcitonin (PCT) have become established surrogate markers for infections [4,5,6,7].

IL 6 has gained attention as an additional marker due to its rapid kinetics and short half-life. It is among the fastest-responding inflammatory biomarkers, rising 24 to 48 h before clinical signs of inflammation [8] and peaking approximately 6 h post-exposure [9]. In comparison, C-reactive protein (CRP) rises 10–12 h after stimulation, peaks after approximately 36–50 h, and declines with a half-life of approximately 19 h once the inflammatory focus is controlled [4]. For CRP, a strong predictive value was reported for ventilator-associated pneumonia (VAP) in both surgical and medical ICU patients with an area under the receiver operating characteristic curve (AUROC) of 0.912 (95% CI 0.847–0.950) when measured 72 h after intubation [10]. Procalcitonin (PCT) rises, similar to IL-6, within 4–6 h after stimulation, reaching peak concentrations between 12 and 24 h post-exposure; it helps distinguish bacterial from viral infections with a sensitivity of approximately 92% and a specificity of 73%, although its diagnostic performance varies depending on the causative pathogen. Even after severe trauma, particularly involving abdominal organs, an increase in PCT levels has been documented [8,11]. Among several evaluated biomarkers, IL-6 was the only one capable of differentiating between microbiologically confirmed and merely suspected cases of VAP [12]. However, the clinical relevance of IL-6 in diagnosing infections in the ICU remains controversial, as its elevation can result from a wide range of inflammatory processes. Despite putative advantages, other studies have demonstrated only a moderate diagnostic value of IL-6 in differentiating confirmed infections from suspected infections [13,14,15,16].

In former studies, it was assumed that PCT-guided therapy may reduce mortality at 28 days and after one year [17,18] and decrease antibiotic consumption but is not necessary with a shortened ICU length of stay [19]. Our working assumption was that regular IL-6 use may be associated with a shorter ICU length of stay (LOS) and antibiotic consumption by facilitating faster discontinuation. Therefore, the aim of this study was to explore whether the routine use of IL-6, compared with CRP/PCT, is associated with shorter ICU LOS (primary outcome) and ventilation hours, ICU mortality, and antibiotic use (secondary outcomes).

## 2. Materials and Methods

The Clinical Setting: This retrospective observational study was conducted at the interdisciplinary ICU and intermediate care unit, collectively referred to as the ICU in the following, of the Helios Dr. Horst-Schmidt-Kliniken in Wiesbaden, Germany. IL-6 was routinely used every day as a marker for infection from 2019 until 31 March 2022. Due to institutional and economic considerations, IL-6 testing was discontinued and replaced by the combination of CRP and PCT, jointly referred to here as CRP/PCT, from 1 April 2022 onward.

Patients with an end of ICU stay between 1 April 2021, and 31 March 2023, were included. Exclusion criteria were age < 18, ≤24 h hours of ventilation, and pregnancy. Because COVID-19 elicits a strong inflammatory response and there was no prior experience with this condition at that time, patients were excluded from specific analyses as indicated. In a pilot cohort, patients with an end of stay between 1 April and 31 May 2021, were assigned to the IL-6 group, and between 1 April and 31 May 2022, to the CRP/PCT comparison group. For confirmatory analyses in a larger cohort, the IL-6 group was extended by patients with an end of stay by 31 March 2022, and the CRP/PCT group by 31 March 2023. The two comparison periods in each cohort were chosen in consecutive years to minimize the effect of seasonal variation, particularly in respiratory infections.

Data on demographics, underlying medical conditions, laboratory values, vital signs, treatment parameters, initiation and duration of antimicrobial therapies, and ICU mortality were retrieved from the clinical information management system ICM (Dräger^®^), the anesthesia and ICU documentation system MetaVision (IMD Soft^®^), and the hospital information system (SAP^®^). Days of antimicrobial therapy (DOT) were defined as the sum of calendar days on which a patient received at least one dose of a given antimicrobial agent. During combination therapy, one DOT was counted per agent and calendar day. The WHO-defined defined daily dose (DDD) and recommended daily dose (RDD) were used to quantify antibiotic consumption. DDD represents the assumed average adult maintenance dose for the main indication, and RDD reflects guideline-based daily doses for clinical indications. As measures of disease severity, we utilized the SAPS II score and the German intensive care complex treatment (ICCT) code, which is based on the modified SAPS II score of the first 24 h after admission, the need for mechanical ventilation and its duration, acute renal replacement therapy (intermittent hemodialysis or continuous renal replacement therapy), and extracorporeal membrane oxygenation (ECMO) [20,21].

The primary outcome was the ICU LOS. Secondary outcomes included mechanical ventilation duration, ICU mortality, and antimicrobial consumption.

Statistical Analysis: Depending on distribution, variables were compared using t tests or Mann–Whitney U tests. Metric variables are indicated as the mean (SD). Cohen’s d (d) or Cramer’s V (V) were used to determine the corresponding effect size [22]. Effect sizes were interpreted using conventional thresholds: small (Cohen’s d ≈ 0.2, rank-biserial r ≈ 0.1, Cramer’s V ≈ 0.1), medium (d ≈ 0.5, r/V ≈ 0.3), and large (d ≥ 0.8, r/V ≥ 0.5). Categorical variables were analyzed using chi-square tests. A *p* value of less than 0.05 was considered statistically significant. Multivariable regression was performed for the ICU LOS for c variables age, sex, and ICCT categories. Antibiotic consumption (DDD and RDD) was modeled using Poisson regression to estimate rate ratios between the IL-6 and CRP/PCT groups. The statistical analysis was performed with JASP 0.18.2, and matching and graph visualization were performed with Julius.ai 1.1 Max (Caesar Labs, Inc., San Francisco, CA, USA).

## 3. Results

### Pilot Cohort

**Figure A1:**
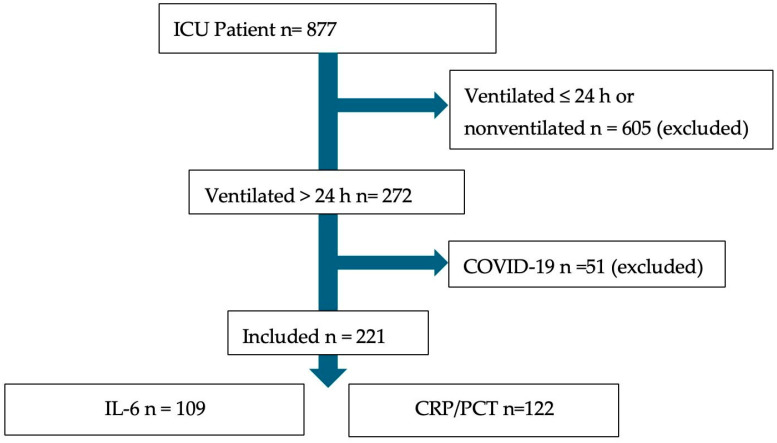
Flowchart of the pilot cohort.

Baseline: For the pilot cohort, a total of 221 ICU patients met the inclusion criteria, with 109 patients in the IL-6 group and 112 in the CRP/PCT group (Appendix A Figure A1). There was no statistically significant difference in disease severity between the groups, as indicated by median SAPS II scores of 36 vs. 34 (*p* = 0.351, r = 0.073). Likewise, no significant differences were found in most baseline clinical parameters, except for a lower Horowitz index and bicarbonate levels in the CRP/PCT group. Regarding comorbidities, the CRP/PCT group had a higher prevalence of peripheral arterial disease (16.96% vs. 7.34%, *p* = 0.039, V = 0.029). Details are given in Table 1 and Appendix A, Table A1 (baseline and SAPS II) and Table A2 (anamnestic characteristics).

Primary outcome: There were no significant differences between the two groups with respect to the mean ICU LOS (4.18 [4.12] vs. 4.29 [4.94] days, *p* = 0.419, r = 0.068) or ICU mortality (21.10% vs. 16.07%, *p* = 0.3884, V = 0.025). In the overall cohort, the proportion of ventilated patients was significantly higher in the IL-6 group than in the PCT/CRP group (76.14% vs. 53.57%, *p* < 0.001, V = 0.236). However, among patients receiving antimicrobial therapy, this difference was no longer statistically significant (87.1% vs. 78.2%, *p* = 0.201, V= 0.118). Also, the length of ventilation among those patients who were ventilated did not differ significantly between the groups (60.87 [79.70] h vs. 77.95 [89.92] h, *p* = 0.203, r = 0.125). Details are given in Table 2.

Secondary outcome: The proportion of patients receiving any antimicrobial therapy did not differ significantly between groups (56.88% in the IL-6 group vs. 49.10% in the CRP/PCT group, *p* = 0.247, V = 0.078). A similar pattern was observed across different types of antimicrobial therapy—antibiotics (55.96% vs. 48.21%, *p* = 0.248, V = 0.078), antifungals (2.75% vs. 5.36%, *p* = 0.327, V = 0.066), and antivirals (3.67% vs. 1.79%, *p* = 0.389, V = 0.058). The mean days of antimicrobial therapy (DOT) were higher in the IL-6 group (4.07 [6.57]) than in the control group (2.95 [4.51]), but the difference was not statistically significant (*p* = 0.136, r = 0.110). Details are given in Table 2.

**Figure A2:**
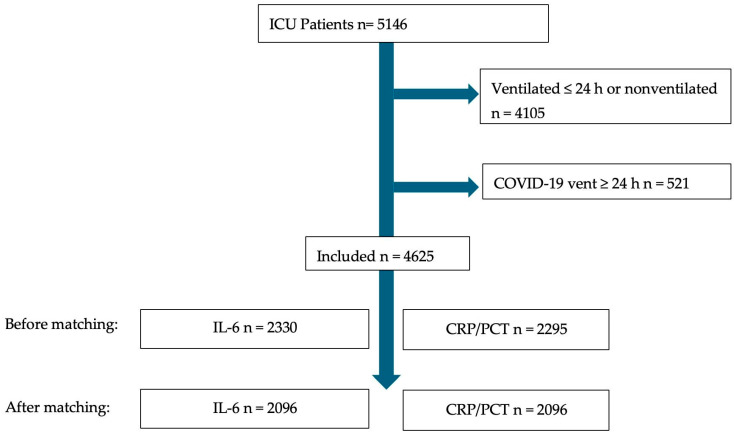
Flowchart of the extended cohort.

Extended Cohort:

Baseline: For the extended cohort, 5146 ICU patients were included, with 2576 in the IL-6 group and 2570 in the PCT/CRP group (Appendix A Figure A2). The disease severity was calculated and compared between the groups using the ICCT. Details are given in Table 3.

We did not find a difference in the distribution of internal and surgical patients (χ^2^(1) = 3.70, *p* = 0.054). Details are given in Appendix A, Table A3.

Likewise, there were no significant differences in age (67.10 [16.02] vs. 67.10 [16.53] *p* = 0.996, d < 0.000). The sex distribution indicated a significant difference between the two cohorts (χ^2^(1, N = 5146) = 5.69, *p* = 0.017, V = 0.033), with more men in the first IL-6 group and more women in the PCT/CRP group. However, the difference is statistically significant but with an effect size of 0.033, which is not of practical relevance.

Primary outcome:

To avoid confounding by COVID-19, 521 COVID-19 patients were excluded. Thereafter, one-to-one matching was performed within each ICCT level, using the leading specialty as the matching variable (e.g., IL-6 neurosurgical patients in ICCT level 3 were matched to PCT/CRP neurosurgical patients in ICCT level 3). This led to a sample size of 2096 patients in each group.

Here, mortality did not differ significantly between groups, χ^2^(1, N = 4192) = 1.28, *p* = 0.258, V = 0.017 (327 (15.6%) in the IL-6 group, compared to 354 patients (16.9%) in the PCT/CRP group. Additionally, the hours of ventilation and the ICU length of stay did not differ significantly between the cohorts. Details are given in Table 4.

Additionally, after adjustment for age, sex, and ICCT categories, ICU LOS was not significantly longer in the PCT/CRP in the negative binomial model (IRR 1.05, 95% CI 0.97–1.13). There was no meaningful multicollinearity among these predictors (VIF 1.00–1.02).

Secondary outcome:

For the secondary outcome, also COVID-19 patients were included. Although the IL-6 group had more COVID-19-positive patients than the PCT/CRP group (234 vs. 164), total ICU days were very similar between the groups (1529 vs. 1550 days).

We found higher antibiotic consumption in the IL-6 group than in the PCT/CRP group, both for DDD (16.8% increase, rate ratio RR = 1.17, 95% CI (1.14, 1.19), *p* < 0.001) and RDD (11.6% increase, RR = 1.12, 95% CI (1.09, 1.14), *p* < 0.001). (Table 5).

## 4. Discussion

Our study aimed to determine whether the daily routine use of IL-6 as an infection marker influences the ICU LOS, duration of mechanical ventilation, ICU mortality or antibiotic consumption in ICU patients compared to PCT/CRP.

Primary outcome: The results of our investigation suggest that IL-6, compared to PCT/CRP, did not significantly impact patient outcomes in our ICU setting. Despite the theoretical advantages of IL-6—namely, its rapid rise and fall in serum levels—its routine use did not result in a different outcome concerning ICU LOS, ventilation hours and ICU mortality. Although IL-6 has been associated with disease severity and prognosis in sepsis [14,16], our data indicate that this biomarker provided no additional clinical benefit when applied routinely. Baseline IL-6 and 96-h IL-6 clearance were able to demonstrate some discriminatory ability in mortality risk [23]. In terms of outcomes, mortality was comparable between groups, within the predicted range for the SAPS II score.

Secondary outcome: In our first cohort, we found a trend toward reduced antimicrobial use after IL-6 testing was discontinued. Next, we tested this hypothesis on a much larger cohort, confirming a significant decrease in antimicrobial use. Since we were not able to exclude COVID-19 patients for this latter calculation, the higher antibiotic consumption in the IL-6 group could be explained by the more severe COVID-19 cases in the first time period, accounting for higher ventilation hours within this group. The relationship between COVID-19 severity and interleukin 6 levels is well established [24]. These higher levels might have prompted the treating physicians to initiate or prolong antibiotic therapy. Therefore, we must assume that a significant proportion of the higher antibiotic consumption in the IL-6 group is related to this. Nevertheless, we also observed this trend within the pilot cohort that excluded COVID-19 patients.

One further possible explanation for this trend might be that IL-6 may have led to a more aggressive therapeutic approach when elevated. IL-6 has shown a high sensitivity in discriminating sepsis and septic shock from controls with systemic inflammatory response syndrome (SIRS) [16] but a lag of specificity [13,25]. This lag could promote overtreatment, particularly in heterogeneous ICU populations where sterile inflammation is frequent (e.g., after surgery, trauma, or ischemia–reperfusion injury). Consequently, we may have interpreted IL-6 elevations as infection triggers, resulting in unnecessary or prolonged antimicrobial therapy. In contrast, PCT-guided algorithms have been shown to shorten antibiotic courses when they are implemented in a standardized protocol [11,26]. In our setting, IL-6 values were available daily without a predefined protocol in antibiotic disruption, which might have led to subjective treatment decisions rather than improving stewardship.

Besides biomarker monitoring, microbiological analysis of suitable specimens remains highly valuable, with microbiological cultures still serving as the gold standard for detecting phenotypic antibiotic resistance. However, rapid molecular techniques such as multiplex real-time PCR and next-generation sequencing allow for earlier pathogen identification and may decrease antibiotic exposure, although diagnostic accuracy can differ among pathogens. Emerging technologies, including nanopore sequencing, also offer near real-time bedside diagnostics, potentially supporting more targeted antimicrobial therapy [27,28].

In addition, studies like the STOP-IT Trial (Study to Optimize Peritoneal Infection Therapy) demonstrated that a predefined duration of antimicrobial therapy, independent of biomarker levels, was not inferior in terms of clinical outcomes [29].

Routine laboratory testing is usually performed every day at the same hour, with the same routine markers for every patient. In this setting, the physician in charge makes the decision to prescribe antibiotics. Different factors influence this decision, such as the experience level of the physician or the interpretation standards for biomarkers within the department. Additional factors such as time pressure, requests from patients/carers, diagnostic uncertainty and reliance on evidence-based guidelines and diagnostic tests greatly influence when and how we decide [30,31,32]. One of these factors we have illuminated in this study is the influence of biomarkers. In a previous study, we showed that IL-6 is a favorable marker, outperforming PCT and CRP in differentiating patients with ventilator-associated pneumonia [33]. However, unreflective daily use might lower the threshold for prescribing an antibiotic [32]. Therefore, it might be reasonable to embed the advantages of IL-6 compared to PCT or CRP into protocols. These protocols should specify the timing of the test, which might be supported by artificial intelligence, which has already been shown to reduce unnecessary tests [34,35].

Strengths and Limitations: This study’s strength lies in its two-step design, which combines detailed single-cohort data with a large, system-wide dataset across consecutive years, reducing seasonal and case-mix bias. However, there are several limitations.

First, the study was designed as a retrospective single-center study, which may imply that hospital-specific factors, as well as unrecorded clinical variables, could have influenced treatment decisions. In addition, we did not differentiate between infection types or confirm microbiological findings in the extended cohort. Furthermore, dose–response relationships or thresholds for the initiation of antibiotic therapy were not recorded.

Within the pilot cohort, we found a higher proportion of ventilated patients in the IL-6 group. This was likely reflecting temporal and case-mix differences between the comparison periods. Nevertheless, this represents a potential confounder. However, this imbalance was not reproduced in the extended cohort and was not evident among patients receiving antimicrobial therapy.

ICU LOS represents a multifactorial outcome influenced by factors beyond infection and antimicrobial therapy, including ICU capacity, discharge logistics, and organizational constraints. While the large sample size was intended to mitigate the impact of such variability, residual confounding cannot be excluded. Second, antimicrobial utilization could only be assessed at a general level, as detailed patient-level data on indication, appropriateness, microbiological confirmation, cofounding factors, such as organ replacement therapies, and treatment modifications were not available.

For antibiotic consumption metrics (DDD and RDD), exclusion of COVID-19 patients was not feasible without introducing substantial selection bias. Given the known impact of COVID-19 on inflammatory markers and antimicrobial use, this represents a relevant confounding factor. However, when comparing the total ICU length of stay of COVID-19 patients between the IL-6 and PCT/CRP groups, only a minimal difference of 11 days was observed. Nevertheless, these analyses were considered exploratory and should be interpreted with caution. Statistically, this study employed an exploratory, descriptive design. To support a large sample size, we also did not perform individual-level propensity score matching, which might have reduced the risk of selection bias. However, we could demonstrate group-level homogeneity. Additionally, no formal adjustment for multiple testing was applied to secondary outcomes, as these analyses were exploratory and the endpoints were conceptually related. The results should be interpreted accordingly. Future studies should evaluate IL-6 within protocolized antimicrobial stewardship algorithms and its advantages over CRP and/or PCT with respect to clinically relevant outcomes.

## 5. Conclusions

In this exploratory ICU study, routine IL-6 monitoring was not associated with improved clinical outcomes but may be associated with increased antimicrobial use, emphasizing the need for structured, stewardship-guided implementation of biomarker-based decision-making.

Therefore, future prospective studies should focus more on antibiotic utilization, including detailed patient-level data on antimicrobial therapy, microbiological confirmation, and relevant clinical confounders, while evaluating standardized biomarker-guided algorithms within antimicrobial stewardship frameworks.

Future considerations might include integrating reliable biomarkers and AI-driven decision pathways, determining when to obtain a laboratory sample, and determining whether to initiate antibiotic therapy to support bedside physicians in their decision-making. Nevertheless, this should always be embedded within a structured stewardship programme.

## Figures and Tables

**Table 1:** Baseline characteristics of pilot cohort.

Characteristic	IL-6 n = 109	CRP/PCT n = 112	*p* Value (95% CI)
Age (years) as the mean (SD)	69.72 (16.82)	66.08 (17.09)	0.112 (−0.862–8.131)
Sex			
Male	63 (57.7%)	76 (67.85%)	
Female	46 (42.21%)	36 (32.15%)	0.128
SAPS II (points) as the mean (SD)	38.61 (18.53)	36.54 (15.12)	0.3510 (−2.418–6.539)
Chronic disease			
None	102 (93.58%)	105 (93.75%)	
Metastatic cancer	6 (5.50%)	4 (3.57%)	
Hematologic malignancy	1 (0.92%)	3 (2.68%)	
AIDS	0 (0.0%)	0 (0.0%)	0.496
Reason for admission			
Elective surgery	9 (8.26%)	9 (8.03%)	
Medical	71 (65.14%)	70 (62.50%)	
Emergency surgery	29 (26.61%)	33 (29.46)	0.437

Values are presented as the mean (SD) or as absolute and relative frequencies. 95% CIs refer to the mean difference. SAPS II = Simplified Acute Physiology Score II. AIDS = acquired immunodeficiency syndrome. Statistically significant *p* values are italicized.

**Table 2:** Primary and secondary outcomes of patients with and without IL-6 measurements.

Primary Outcome	IL-6 (n = 109)	CRP/PCT (n = 112)	*p* Value (95% CI)
Length of stay (days)	4.18 (4.12)	4.30 (4.94)	0.856 (−1.319–1.096)
Proportion of ventilated patients	83/109 (76.14%)	60/112 (53.57%)	*<0.001*
Duration of ventilation (h)	46.35 (74.18)	41.76 (76.30)	0.651 (−15.37–24.55)
Mortality	23 (21.10%)	18 (16.07%)	0.336
Secondary outcome			
Patients with antimicrobial therapy	62 (56.88%)	55 (49.10%)	0.247
– Antibiotic therapy	61 (55.96%)	54 (48.21%)	0.249
– Antimycotic therapy	3 (2.75%)	6 (5.36%)	0.327
– Antiviral therapy	4 (3.67%)	2 (1.79%)	0.389
Mean Days of antimicrobial therapy (DOT)	4.07 (6.57)	2.95 (4.50)	0.137 (−0.363–2.617)
Days of antimicrobial therapy (DOT) for treated patients	7.16 (7.34)	6.00 (4.80)	0.320 (−1.142–3.465)

Values are presented as the mean (SD) or as absolute and relative frequencies. 95% CIs refer to the mean difference. Statistically significant *p* values are italicized.

**Table 3:** Distribution of patients across ICCT categories by cohort.

Group	0	1–2	3–5	≥6
IL-6	1797 (69.8%)	499 (19.4%)	208 (8.1%)	72 (2.8%)
PCT/CRP	1789 (69.7%)	513 (20.0%)	189 (7.4%)	77 (3.0%)

χ^2^(3) = 1.28, *p* = 0.735, V = 0.016. Values are given as absolute numbers (percentages).

**Table 4:** Descriptive statistics and independent samples *t* tests for IL-6 versus PCT/CRP group for the extended cohort.

Variable	IL-6 (n = 2096) Mean (SD)	PCT/CRP (n = 2096) Mean (SD)	*p* Value
Hours of ventilation	28.75 (98.60)	26.49 (97.96)	0.456
ICU LOS (days)	3.82 (6.35)	4.11 (7.63)	0.181

Values are given as the means with standard deviations in parentheses. Student’s *t* test was used.

**Table 5:** Antibiotic consumption within the IL-6 group compared to the PCT/CRP group.

	IL-6	PCT/CRP
Total patient days	11,799	13,256
DDD per 100 PD	153	131
RDD per 100 PD	125	112
Total DDD	18,052.47	17,365.36
Total RDD	14,748.75	14,846.72

DDD = defined daily dose; RDD = recommended daily dose; PD = patient days.

## Data Availability

The data presented in this study are not publicly available due to legal and ethical restrictions related to the use of sensitive patient data and data protection regulations. Anonymized or aggregated datasets may be made available from the corresponding author upon reasonable request and with appropriate justification and compliance with applicable data protection laws.

## Abbreviations

The following abbreviations are used in this manuscript:

AIDS	acquired immunodeficiency syndrome
AUROC	area under the receiver operating characteristic curve
CI	confidence interval
COPD	chronic obstructive pulmonary disease
DDD	defined daily dose
DOT	days of therapy
ECMO	extracorporeal membrane oxygenation
ICU	intensive care unit
ICCT	intensive care complex treatment
IL-6	interleukin-6
IRR	incidence rate ratio
LOS	length of stay
PCT	procalcitonin
PD	patient days
RDD	recommended daily dose
RR	rate ratio
SAPS II	Simplified Acute Physiology Score II
SD	standard deviation
SIRS	systemic inflammatory response syndrome
VAP	ventilator-associated pneumonia
VIF	variance inflation factor
WHO	World Health Organization

## Appendix A

**Table A1:** Baseline SAPS II characteristics of the pilot cohort.

Characteristic	IL-6 (n = 109)	PCT/CRP (n = 112)	*p* Value
SAPS II (points) *	36 (17.00)	34 (20.23)	0.3510
Age (years) *	74 (20.00)	68 (23.23)	0.0640
Sex			
– Male	63 (57.79%)	76 (67.85%)	
– Female	46 (42.21%)	36 (32.15%)	0.1282
Heart rate (beats/min)			
– 70–119	33 (30.28%)	32 (28.57%)	
– 40–69	43 (39.45%)	35 (31.25%)	
– 120–159	26 (23.85%)	32 (28.57%)	
– >160	3 (2.75%)	8 (7.14%)	
– <40	4 (3.67%)	5 (4.46%)	0.4337
Systolic blood pressure (mmHg)			
– 100–199	40 (36.70%)	36 (32.14%)	
– >200	3 (2.75%)	4 (3.57%)	
– 70–99	61 (55.96%)	61 (54.46%)	
– <70	5 (4.59%)	11 (9.82%)	0.4640
Temperature (°C)			
– <39	100 (91.74%)	104 (92.86%)	
– ≥39	9 (8.26%)	8 (7.14%)	0.8049
Horowitz index			
– No ventilation	29 (26.61%)	56 (50.00%)	
– ≥200	54 (49.54%)	34 (30.36%)	
– 100–199	21 (19.27%)	16 (14.29%)	
– <100	5 (4.59%)	6 (5.36%)	*0.0031* *
Urea (mg/dL)			
– <60	74 (67.89%)	78 (69.64%)	
– 61–179	31 (28.44%)	28 (25.00%)	
– >180	4 (3.67%)	6 (5.36%)	0.7345
Urine output (mL/24 h)			
– >1000	74 (67.89%)	78 (69.64%)	
– 500–1000	25 (22.94%)	26 (23.21%)	
– <500	10 (9.17%)	8 (7.14%)	0.8579
Sodium (mmol/L)			
– 125–144	90 (82.57%)	94 (83.93%)	
– >145	18 (16.51%)	14 (12.50%)	
– <125	1 (0.92%)	4 (3.57%)	0.3093
Potassium (mmol/L)			
– 3.0–4.9	100 (91.74%)	98 (87.50%)	
– ≤3.0	2 (1.83%)	3 (2.68%)	
– ≥5.0	7 (6.42%)	11 (9.82%)	0.6200
Bicarbonate (mmol/L)			
– ≥20	81 (74.31%)	98 (87.50%)	
– 15–19	21 (19.27%)	10 (8.93%)	
– <15	7 (6.42%)	4 (3.57%)	*0.0429 **
Bilirubin (mg/dL)			
– <4.0	105 (96.33%)	108 (96.43%)	
– 4.0–5.9	0 (0.0%)	2 (1.79%)	
– ≥6.0	4 (3.67%)	2 (1.79%)	0.2633
Leukocytes (cells ×10^9^/L)			
– 1.0–19.9	91 (83.49%)	100 (89.29%)	
– ≥20.0	17 (15.60%)	11 (9.82%)	
– <1.0	1 (0.92%)	1 (0.89%)	0.4340
Chronic disease *			
– None	102 (93.58%)	105 (93.75%)	
– 1	6 (5.50%)	4 (3.57%)	
– 2	1 (0.92%)	3 (2.68%)	
– 3	0 (0.0%)	0 (0.0%)	0.4958
Reason for admission **			
– 1	9 (8.26%)	9 (8.03%)	
– 2	71 (65.14%)	70 (62.50%)	
– 3	29 (26.61%)	33 (29.46%)	0.4373

Values are presented as median (interquartile range) or as absolute and relative frequencies. SAPS II = Simplified Acute Physiology Score II. * Chronic disease: 1 = metastatic cancer, 2 = hematologic malignancy, 3 = acquired immunodeficiency syndrome (AIDS). ** Reason for admission: 1 = elective surgery, 2 = medical, 3 = emergency surgery. Statistically significant *p* values are italicized.

**Table A2:** Anamnestic characteristics of patients with and without IL-6 measurements.

Characteristic	IL-6 (n = 109)	PCT/CRP (n = 112)	*p* Value
Chronic renal failure	15 (13.76%)	16 (14.29%)	0.911
COPD	14 (12.84%)	8 (7.14%)	0.157
Asthma	6 (5.50%)	6 (5.36%)	0.961
Arterial hypertension	64 (58.72%)	59 (52.68%)	0.366
Coronary artery disease	23 (21.10%)	32 (28.57%)	0.199
Acute myocardial infarction	12 (11.01%)	21 (18.75%)	0.106
Peripheral arterial disease	8 (7.34%)	19 (16.96%)	*0.029*
Heart failure	20 (18.35%)	32 (28.57%)	0.073
Neoplasia	23 (21.10%)	24 (21.43%)	0.953
Cerebral ischemia	16 (14.68%)	12 (10.71%)	0.376
Intracerebral hemorrhage	4 (3.67%)	5 (4.46%)	0.765
Epilepsy	5 (4.59%)	5 (4.46%)	0.965
High-dose steroids	0 (0.0%)	0 (0.0%)	—
Diabetes mellitus	27 (24.77%)	25 (22.32%)	0.668
Dyslipoproteinemia	16 (14.68%)	17 (15.18%)	0.917
Nicotine abuse	12 (11.01%)	23 (20.54%)	0.052

Values are presented as absolute and relative frequencies. COPD = chronic obstructive pulmonary disease.

**Table A3:** Comparison of Internal vs. Surgical Cases Across ZK Groups.

ICCT Group	n (Total)	Internal Medicine IL-6	Surgical Medicine IL-6	Internal Medicine PCT/CRP	Surgical Medicine PCT/CRP	χ^2^	*p*	df	Cramér’s V
0	3586	759	1038	868	921	14.02	<0.001	1	0.063
1–2	1012	326	173	320	193	0.83	0.362	1	0.029
3–5	397	114	94	90	99	1.77	0.183	1	0.067
6	149	40	32	28	49	4.78	0.029	1	0.179

C1 = Cohort 1; C2 = Cohort 2. Values are absolute numbers. Chi-square tests were used to compare internal vs. surgical cases.
